# Skeletal Muscle Oxygen Dynamics during Rehabilitation of Children with Congenital Heart Disease: A Comparative Study of Two Cases

**DOI:** 10.1298/ptr.25-E10341

**Published:** 2025-05-30

**Authors:** Yusuke KAWAMURA, Hironori MATSUHISA, Yuki NONAKA, Tetsuya FUKUDA, Yosuke IKEDA, Kazuyuki TABIRA

**Affiliations:** 1 Department of Rehabilitation, Kobe Children’s Hospital, Japan; 2 Department of Cardiovascular Surgery, Kobe Children’s Hospital, Japan; 3 Graduate School of Health Science, Kio University, Japan

**Keywords:** Congenital heart disease, Rehabilitation, NIRS

## Abstract

Objectives: The need for early postoperative rehabilitation in patients with congenital heart disease (CHD) is increasing. However, rehabilitation settings in the pediatric intensive care unit (PICU) are usually determined subjectively by therapists. To address the lack of objective measurements, we sought to determine the effectiveness of near-infrared spectroscopy (NIRS) in evaluating skeletal muscles during rehabilitation of patients in the PICU. This case series aimed to clarify the characteristics of skeletal muscle oxygenation during exercise in 2 postoperative patients with CHD. Methods: The participants were two 6-month-female infant: one had undergone the Yasui operation (Case 1), and the other had undergone a bidirectional Glenn anastomosis (Case 2). Vital signs and tissue oxygen saturation (StO_2_) were measured during each exercise task, and the muscle oxygen extraction ratio (MOER), an index of the intramuscular oxygen extraction rate, was calculated. The results were compared between the two cases. Results: Case 1 showed no significant changes in vital signs, StO_2_, or MOER, whereas Case 2 had low oxygen saturation at rest and low StO_2_ during the exercise tasks. MOER increased during the exercise tasks. Conclusions: The results showed that sitting did not impose a strong cardiopulmonary load on postoperative patients. However, in children with cyanotic cardiac disease, such as in Case 2, skeletal muscle oxygenation should be considered, and NIRS monitoring may be useful for safely performing rehabilitation.

## Introduction

Approximately 10000 children are born with congenital heart disease (CHD) annually in Japan^1)^. Since the 1990s, the treatment of children with CHD has become safer, even for severe cases, and surgery is now performed at early neonatal and infant stages; as a result, the age at surgery has decreased and the indications for surgery have expanded^2)^. In the 2000s, the standardization of CHD surgical treatment progressed, and the results of neonatal surgery greatly improved^3)^. With advances in cardiac surgical techniques and intensive care, the long-term survival of patients with CHD has improved^4)^, and physical therapy is now actively provided to postoperative patients with CHD. However, many patients still show developmental delays. In addition, it has become clear that physical function declines from the time of admission to the pediatric intensive care unit (PICU), increasing the need for early rehabilitation^5–7)^.

In the rehabilitation of patients with CHD, it is necessary to consider each patient’s pathophysiology and manage their individual risks. Japanese guidelines for the rehabilitation of patients with cardiac disease also emphasize the importance of exercise therapy, but recommend that exercise therapy be carefully administered to patients aged <6 years and for whom cardiopulmonary exercise testing is difficult to perform^8)^. Unlike adults, children may not be able to appropriately express their will, such as complaints of fatigue, so subjective assessments, such as observing changes in facial expressions or crying, are required. Therefore, the load setting for rehabilitation in the PICU is usually determined subjectively by a physical therapist.

Recently, several studies have reported on the use of near-infrared spectroscopy (NIRS) to measure oxygen saturation in the skeletal muscles^9–12)^. NIRS can noninvasively evaluate the response of skeletal muscle blood flow and oxygen consumption during exercise, and it can be used to determine the effectiveness of rehabilitation^9–13)^. In pediatric cardiac surgery, NIRS is used to evaluate perioperative systemic perfusion in high-risk patients, such as those with hypoplastic left heart syndrome (HLHS)^14)^. NIRS monitoring may assist in dose setting during rehabilitation of patients with CHD in the PICU. However, few studies have examined skeletal muscle oxygen saturation in infants using NIRS. The purpose of this case series was to characterize skeletal muscle oxygen dynamics during exercise loading in 2 patients with postoperative CHD.

## Case Presentations

This study was approved by the Kobe Children’s Hospital Institutional Ethics Committee (No:2024-114) and conducted in accordance with the Declaration of Helsinki. The patients' parents provided informed consent to participate in this study.

In this report, NIRS evaluation was performed in 2 pediatric patients with different circulatory physiologies. One patient had undergone biventricular repair, resulting in complete resolution of cyanosis, while the other had a single-ventricle physiology in the interstage period, with persistent cyanosis.

### Case 1

A 6-month-female infant had congenital aortic stenosis (AS), coarctation of the aorta, and a ventricular septal defect (VSD). She had two adequately sized ventricles and underwent a staged biventricular repair (Yasui operation). This procedure consisted of aortic arch repair, integration of the small ascending aorta and well-developed proximal main pulmonary artery (Damus–Kaye–Stansel procedure), followed by intraventricular tunneling from the VSD to the pulmonary valve, and conduit placement from the right ventriculotomy to the distal pulmonary artery. Following the operation, the patient achieved biventricular circulation and cyanosis was completely resolved. However, the entire surgery required an extended period of cardiac arrest and cardiopulmonary bypass support. The chest was closed on the 3rd postoperative day, and the patient was extubated on the 8th postoperative day. Postoperative rehabilitation, according to the early mobilization protocol (Table 1), began on the 6th postoperative day. Measurements of skeletal muscle oxygenation during rehabilitation were performed on the 13th postoperative day (Level 2 exercise task). Her oxygen saturation (SpO_2_) was 100% with 1.0 L/min of nasal oxygen administration.

**Table 1.** PICU early mobilization protocol

Level 1	Gradual head-up (target 30°)	FiO_2_ >60%, PEEP >8
	Body crossings every 2 h	Intubation/difficult ventilation, post-tracheostomy (until first exchange)
	Implementation of positioning	Use of catecholamines
		SBS: −2 to −3
		Drain, ICP, EVD (not clampable)
Level 2	Head-up 45° or end-sitting position	FiO_2_ <60%, PEEP <8 (intubated)
		FiO_2_ >60%, NPPV
		SBS >−1
		CV, drain/EVD (can be clamped)
Level 3	Standing or wheelchair (baby carriage)	NPPV <60%, HFNC
	Baby couch/carrier	No intubation tube
Level 4	Wheelchair	Oxygen cannula/mask
	Walking, toilet walking	No arterial line

CV, central venous catheter; EVD, external ventricular drain; FiO_2_, fraction of inspiratory oxygen; HFNC, high-flow nasal cannula oxygen therapy.changes; ICP, intracranial pressure; NPPV, noninvasive pressure ventilation; PEEP, positive end expiratory pressure; PICU, pediatric intensive care unit; SBS, State Behavioral Scale

### Case 2

A 6-month-old female infant was diagnosed with HLHS, and the Norwood procedure was performed. The patient underwent a bidirectional Glenn procedure at 6 months of age. Postoperatively, the patient’s oxygenation stabilized. Mild cyanosis persisted due to a right-to-left shunt of venous blood from the lower body. The patient was extubated on the 1st postoperative day, and her respiratory condition remained stable thereafter. Postoperative rehabilitation following our early mobilization protocol commenced on the 2nd postoperative day. Skeletal muscle oxygenation during rehabilitation was measured on the 6th postoperative day (for a Level 2 exercise task). Her SpO_2_ was 80% with 2.0 L/min of nasal oxygen administration.

The PICU early mobilization protocol advocated by Wieczorek et al. is detailed in Table 1^15)^. Level 1 patients receive a continuous catecholamine infusion and are intubated. In these cases, the interventions focus on joint mobilization, respiratory physiotherapy, and changes in bed position. Exercise loads are introduced only after patients progress to Level 2.

### Assessment of vital signs and skeletal muscle oxygenation during exercise loading

**Fig. 1.**
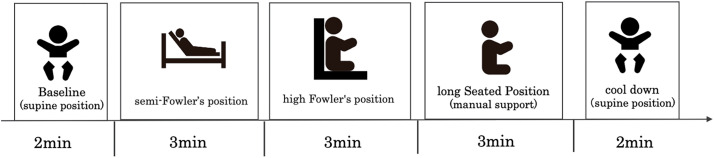
Exercise tasks.

The exercise task corresponds to Level 2 of the early bed release protocol. The patient was placed in a resting supine position for 2 min as baseline, and the exercise task was then divided into 3 phases: semi-Fowler’s position, high Fowler’s position, and sitting with manual support (Fig. 1). Each exercise phase was performed for 3 min. After completion of the exercise task, the patients were again placed in the supine position and allowed to rest for 2 min.

The heart rate (HR), SpO_2_, respiratory rate (RR), and skeletal muscle oxygenation were measured during exercise. Exercise was stopped when HR, systolic blood pressure, or RR increased by more than 20% from baseline value, or if SpO_2_ decreased by 10% or more from baseline.

### Measurement of skeletal muscle oxygen saturation using NIRS

Skeletal muscle oxygenation in the quadriceps muscle was measured using an NIRS machine, INVOS 5100C (Medtronic, Minneapolis, MN, USA) pediatric SOMASENSOR (CV-SPFB/INTL; Medtronic, Minneapolis, MN, USA). The measurement site was located at the midpoint between the anterior superior iliac spine and superior border of the patella^16,17)^. Data acquisition had a temporal resolution of 5–10 s, and in this study, the average values for 30 s before and after each task were calculated and plotted. For signal correction, the INVOS 5100C utilizes light-emitting diodes emitting near-infrared light at 2 wavelengths (730 and 810 nm) and 2 photodiodes to detect transmitted light. By analyzing signal from 2 detectors positioned at different distances from the light source, the device mitigates interference from superficial tissues, such as the skin, and isolates signals from deep tissues, which are the primary target of measurement^18)^.

Tissue oxygen saturation (StO_2_) was used as an index of skeletal muscle oxygenation. The muscle oxygen extraction ratio (MOER) indicates the rate of oxygen extraction in the skeletal muscle, and its usefulness has been documented in previous studies^19,20)^. The MOER was calculated using StO_2_ and SpO_2_, as follows:

$$\text{MOER}＝({\text{SpO}}_{2}-{\text{StO}}_{2})/{\text{SpO}}_{2}$$

StO_2_ is influenced by the oxygenation of both the arterial blood and skeletal muscle, as a decrease in SpO_2_ inevitably leads to a decrease in StO_2_. In contrast, MOER can exclude the effects of arterial oxygenation and specifically reflect the state of peripheral circulation and tissue oxygen consumption. The measurements were averaged over 30 s at the beginning and end of each task.

## Results

**Fig. 2.**
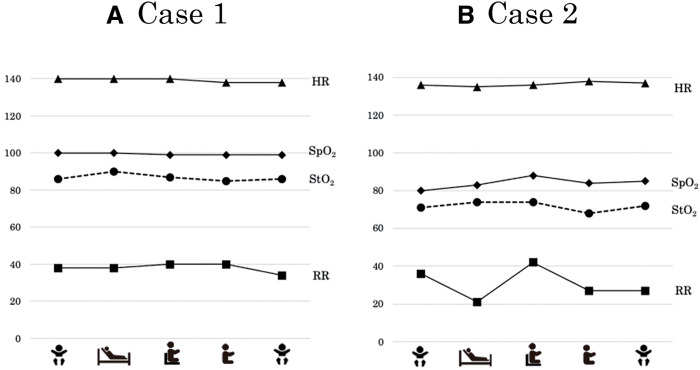
Changes in vital signs and muscle oxygenation indices (A) Case 1 (B) Case 2.

**Fig. 3.**
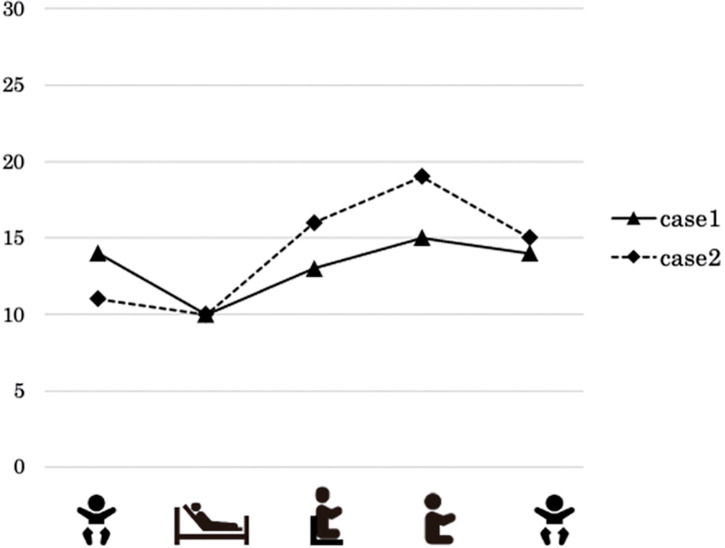
Comparison of MOER (quadriceps). MOER, muscle oxygen extraction ratio

Vital signs for the patient in Case 1 did not fluctuate significantly at rest or during the exercise tasks (Fig. 2A). The average StO_2_ of the quadriceps was 86% at rest, 90% in semi-Fowler’s position, 87% in high-Fowler’s position, and 85% in manually supported sitting. During the 2-min cool-down, the StO_2_ returned to 86%. The MOER was 14% at rest, 10% in the semi-Fowler’s position, 13% in the high Fowler’s position, and 15% in the manually supported sitting position (Fig. 3).

The results of Case 2 are shown in Figure 2B. The HR remained unchanged from rest; SpO_2_ was 83% at rest and increased the most in the high Fowler’s position (88%). The StO_2_ of the quadriceps was 71% at rest, 77% in the semi-Fowler position, 74% in the high-Fowler position, and 68% in the manually supported sitting position. The StO_2_ decreased over time as the intensity of the exercise load increased, such as in the manually supported sitting position. The MOER tended to increase with increasing exercise intensity (Fig. 3).

## Discussion

### Case 1

Coarctation of the aorta (CoA) is a CHD in which the aorta narrows around the arch. Congestive heart failure and shock may occur in severe cases. CoA is often associated with other CHDs, such as VSD^21)^. The patient in Case 1 also had severe AS, which precluded standard aortic arch repair. The Yasui procedure combines the Norwood arch reconstruction (incorporation of a large pulmonary valve into the systemic outflow tract) with a Rastelli operation^22)^. The Yasui procedure can be performed in a staged fashion (neonatal Norwood procedure, followed by a Rastelli-type operation). In infants, after the first stage of surgery (Norwood procedure), cyanosis can remain to some extent. Cyanosis completely resolved after the second stage of surgery (Rastelli operation) for the patient in the first case. In Case 1, as shown in Figure 2A, SpO_2_ during the exercise task was maintained at almost 100%, and StO_2_ remained nearly unchanged during the exercise task. Therefore, the oxygen supply was considered sufficient to increase oxygen consumption during the task. The MOER fluctuated slightly, ranging from 10% to 15%, suggesting that the additional arterial blood supply was sufficient to meet the increase in oxygen consumption. In addition, no changes in RR or HR were observed, suggesting that the intensity of the exercise task may have been too weak.

### Case 2

The Norwood procedure is a life-saving surgery performed during the neonatal period in patients with HLHS. Following the Norwood procedure, patients with HLHS are managed with single ventricles and undergo a bidirectional Glenn procedure as a stage 2 surgery. After this procedure, the patient’s cyanosis partially improved, but SpO_2_ remained at 80%–90%. The patient’s SpO_2_ of 80%–90% at rest and during the exercise task was acceptable for a patient who underwent the bidirectional Glenn procedure. However, this value was significantly lower than that in Case 1. The decrease in StO_2_ may reflect not only an increase in oxygen consumption but also a limited oxygen reserve during the exercise task. By contrast, MOER increased from approximately 10% to 20% during exercise as a compensatory response. This rise in MOER in Case 2 may indicate an insufficient oxygen supply. The observed decrease in StO_2_ during exercise suggests that the reduced oxygen availability in the tissues may have been complemented by an increase in oxygen extraction.

Impaired systemic oxygen delivery and limited exercise capacity have been reported in patients with single ventricle and Fontan circulation^23)^. In particular, those with right ventricular dominance, such as HLHS patients, are associated with poorer functional outcomes. Although data on exercise capacity in children following Glenn procedure are limited, an inadequate increase in cardiac output and oxygen delivery during exercise may have led to a compensatory rise in MOER in Case 2. However, despite this compensatory mechanism, the changes in RR and HR remained relatively small, suggesting that the actual physiological load might not have been as high as expected.

### Comparison of 2 cases

The major differences between the two cases were that SpO_2_ and StO_2_ were lower and MOER was higher in Case 2. This may be due to the lower SpO_2_ in Case 2 because of the residual right-to-left shunting of venous blood and a lower HR response during loading. In addition, it is thought that oxygen consumption in exercising muscles is maintained by enhanced oxygen extraction capability as a response to impaired oxygen supply.

A previous study showed that the quadriceps muscle plays an important role in the control of sitting posture in infants^24)^. Therefore, quadriceps muscle activity is thought to occur during these exercise tasks. In healthy adults, sitting in an unstable seat increased muscle activity in the trunk and thighs^25)^. In the material-supported sitting position, the contact surface was large and stable, directly contrasting the manual sitting position. Therefore, the manual sitting posture requires higher muscle activity and oxygen consumption.

In Case 1, the StO_2_ and MOER values did not change significantly, reflecting sufficient oxygen supply in response to the exercise task. In contrast, it was speculated that the increase in MOER in Case 2 might be a compensatory response to insufficient oxygen supply during the exercise load. Her SpO_2_ did not change during exercise, indicating that the insufficient oxygen supply was due to an impaired circulatory response to increased oxygen demand rather than impaired oxygenation of arterial blood.

The small changes in HR and RR in both cases suggest that the exercise load intensities were relatively low. However, Case 2 responded by increasing the MOER, indicating that the regional skeletal muscles were significantly stressed and that regional perfusion was insufficient. Unlike adults, infants are unable to clearly express their perception of exercise intensity during rehabilitation. Therefore, multiple parameters should be monitored to ensure patient safety, as relying solely on SpO_2_ may lead to misjudgment of exercise intensity limits. This concern is particularly relevant in the acute postoperative phase of CHD, where excessive exercise load may result in sudden clinical deterioration. Previous studies in adults have demonstrated that assessing the balance between oxygen supply and demand during exercise can enhance safety^26)^. Therefore, NIRS is considered a valuable tool for evaluating this balance in pediatric patients. StO_2_ monitoring and MOER measurements may contribute to improving the safety of postoperative rehabilitation in children with CHD.

This case report had some limitations. In this study, a comparative study of these 2 cases was conducted. However, the developmental effects in each case were not considered since the preoperative level of motor development was not assessed. StO_2_ was compared by performing a sitting task as the exercise task; however, the effect of blood flow rate due to postural changes could not be excluded. It is also possible that the child’s body movements, mood, and other factors affected the physiological values while performing the task.

## Conclusions

Vital signs and skeletal muscle oxygenation during the rehabilitation of children with CHD were investigated and compared in 2 different cases. Vital signs fluctuated slightly during the early weaning protocol (sitting task) currently implemented in the PICU, indicating that the protocol does not exert a strong respiratory or circulatory load on postoperative patients. In contrast, in Case 2 (cyanotic heart disease), a more significant decrease in StO_2_ and an increase in MOER were observed, suggesting differences in postoperative hemodynamics. Although the sample in this study is too small to draw definitive conclusions, NIRS may be a valuable tool for establishing a safe zone during postoperative rehabilitation after pediatric heart surgery. Further studies are warranted to validate these findings.

## Acknowledgments

We sincerely thank the patients and their families for their participation in this study. We deeply appreciate their valuable time and willingness to contribute, despite their busy schedules.

## Funding

This research did not receive any specific grant from funding agencies in the public, commercial, or not-for-profit sectors.

## Conflicts of Interest

The authors declare no conflicts of interest.
